# Comparing predictive risk to actual presence of coronary atherosclerosis on coronary computed tomography angiography

**DOI:** 10.1016/j.ahjo.2024.100493

**Published:** 2024-12-06

**Authors:** Emma Playford, Simon Stewart, Gerard Hoyne, Geoff Strange, Girish Dwivedi, Christian Hamilton-Craig, Gemma Figtree, David Playford

**Affiliations:** a The University of Notre Dame Australia, Fremantle, WA, Australia; b Institute for Respiratory Health, QEII Medical Centre, Nedlands, WA, Australia; c Harry Perkins Institute for Medical Research, University of Western Australia, Australia; d Fiona Stanley Hospital, Perth, Australia; e The Prince Charles Hospital, Brisbane, Australia; f Royal North Shore Hospital, Sydney, New South Wales, Australia; g Kolling Institute, University of Sydney, Sydney, New South Wales, Australia

**Keywords:** Atherosclerosis, Coronary computed tomography angiography, Coronary artery disease, Cardiovascular risk scoring

## Abstract

**Background:**

There is limited data showing the predictive accuracy of traditional cardiovascular risk scores (CVRS) to predict asymptomatic coronary artery disease (CAD) determined by coronary computed tomography angiography (CCTA).

**Methods:**

Asymptomatic individuals without known CAD undergoing a screening CCTA and sufficient data to calculate their CVRS, were extracted retrospectively. Atherosclerosis was extracted using natural language processing of the CCTA report, including the coronary artery calcium score (CACS) and the extent and severity of CAD. Absence of atherosclerosis was defined as both zero plaque and zero CACS, and atherosclerosis was defined as low, moderate, or extensive by location and extent of plaque-burden. CVRS was categorized as high (>15 %), moderate (10–15 %), low (1–9 %) and “zero” (<1 %) risk.

**Results:**

828 individuals (median age 58.6, IQR = 52.0, 65.3 years, 57 % male) met inclusion criteria, and a zero, low, moderate, and high CVRS was identified in 13, 483, 113 and 219 individuals (8 %, 49 %, 74 %, 66 % male), respectively. Predominantly low plaque-burden atherosclerosis was detected in 548 scans (67 % male). However, of the 137 males and 68 females with extensive atherosclerosis, 47 (34 %) and 38 (56 %) respectively had low CVRS classification. Overall, 23 % of males and 31 % of females had CAD predicted by CVRS (Monte Carlo: females, *p* = 0.024; males, *p* < 0.001), but there was little to no agreement between CVRS and atherosclerosis burden (Cohen's kappa: males, *κ* = 0.149; females, *κ* = 0.096).

**Conclusions:**

In asymptomatic individuals without known CAD, a low CVRS does not exclude extensive CAD. Newer tools incorporating additional markers may be helpful in risk prediction in such individuals.

## Graphical abstract

**Figure:**
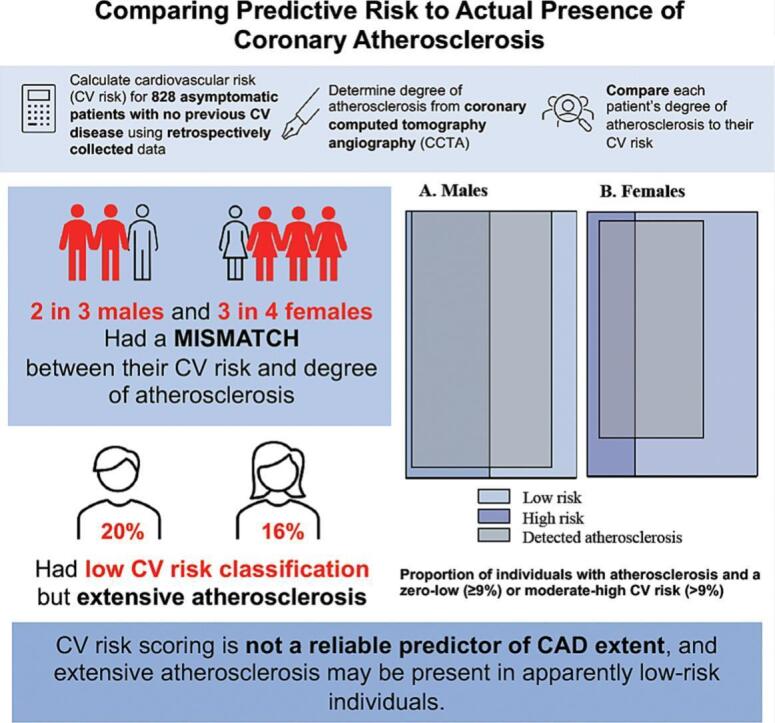

## 1 Introduction

Atherosclerosis is the dominant cause for major cardiovascular events and premature death, with coronary artery disease (CAD) responsible for 10 % of all deaths in Australia [1]. The Australian government recommends routine cardiovascular risk assessment for all individuals over the age of 40, with the goal of implementing lifestyle changes and/or therapeutic treatment in susceptible individuals to reduce the burden of CAD [2]. The Framingham equation, originally devised in 1998 using traditional risk factors to predict cardiovascular risk in asymptomatic individuals [3], has been reported to be used in 44 % of online risk calculators and used by 42 % of general practitioners to inform disease management [4].

However, cardiovascular risk scoring (CVRS) is an imperfect tool. Between 14 and 27 % of first-time myocardial infarction (MI) patients have no standard modifiable risk factors (SMuRFs) [5]. Albarqouni et al. [6] found that the Framingham equation and the pooled cohort risk equation for atherosclerotic cardiovascular disease (PCE-ASCVD) incorrectly predicted 16 % of males and 31 % of females as non-high-risk despite them experiencing a cardiovascular event. Cardiovascular risk estimation errors have been the subject of considerable debate, with the potential to lead to overtreatment (in the absence of significant disease in apparently high-risk individuals) or no treatment (in the presence of severe disease in apparently non-high-risk individuals). This mismatch between apparent risk and actual atherosclerosis burden may increase the risk of a potentially fatal, but preventable, coronary event [5].

Evaluation of CAD plaque burden can be enhanced by coronary artery calcium score (CACS) identified by non-contrast CT [7], as increasing Agatston-units of coronary calcium are associated with adverse survival [8]. Further, non-invasive coronary computed tomography angiography (CCTA) adds important prognostic information by demonstrating the presence and extent of CAD [9], and predicts cardiovascular events after adjustment for CVRS [10]. However CCTA is not risk-free and has financial implications, and as such its role in preventative cardiology has not been entirely defined [11].

There is a significant knowledge gap as to whether cardiovascular risk scoring accurately predicts the presence and extent of CAD in asymptomatic, but potentially at-risk, individuals. The aim of this study was to examine asymptomatic individuals (and no prior history of cardiovascular disease (CVD)) undergoing CCTA for cardiovascular risk assessment, to explore the predictive accuracy of CVRS in detecting the presence and extent of pre-clinical CAD.

## 2 Methods

### 2.1 Study design and population

This was a sub-study of a retrospective observational cohort study of patients undergoing CCTA for cardiovascular risk assessment. The University of Notre Dame Human Research Ethics Committee approved this study (project #019171F) and granted a consent waiver due to the low-risk nature.

The initial cohort consisted of all consecutive patients undergoing CCTA at Perth Radiological Clinic (PRC) in Perth, Western Australia, between January 1, 2018 and December 31, 2019. Patients were excluded if they had a Medicare indication for CCTA (symptoms suggestive of CAD, for exclusion of a coronary anomaly, and/or prior to non-coronary cardiac surgery) or a prior CVD diagnosis, if they were outside of the required age range of 35–74 years old for CVRS calculation, or if any required information to calculate CVRS was missing [12].

### 2.2 Data extraction

#### 2.2.1 CCTA data extraction

The radiologist report from the PRC CCTA results was transcribed by AI-based Natural Language Processing (NLP) through the Australian Institute for Machine Learning (AIML) at the University of Adelaide into “Database 2”. The NLP extracted information on CACS, and radiologist-reported coronary artery findings including presence, extent and severity of plaque, calcification, and location (left anterior descending, circumflex, right coronary and left main arteries). The NLP was trained using manually extracted examples and tested on a separate dataset not previously seen by the AI, and two cardiologists manually verified the output in 200 randomly selected scans. For CACS, NLP accuracy was 99.5 %, recall 100 % and precision 99.3 %; for the presence of atherosclerosis, accuracy was 99.5 %, recall 100 % and precision 99.3 %; and severe vs non-severe stenosis had 99.5 % accuracy, recall 98.7 % and precision 95.7 %.

### 2.3 Degree of atherosclerosis

Three categorical variables were obtained from the CCTA and CACS results and classified according to the Coronary Artery Disease-Reporting and Data System 2.0 (CAD-RADS 2) [13]: CACS in Agatston units (classified as no calcium (0), mild (1−100), moderate (101−300) and high (>300) calcium burden), plaque extent (number of abnormal arteries with ≥1 coronary segment involved) and stenosis severity (either severe (≥70 %) or non-severe (<70 %) from the most severe artery). These numerically categorized variables were added together, with the resultant sum categorized as no atherosclerosis and low plaque-burden, moderate plaque-burden and extensive plaque-burden atherosclerosis. A binary categorical variable was created to record atherosclerosis presence (plaque present and/or non-zero CACS) and atherosclerosis absence (no zero plaque and zero CACS).

#### 2.3.1 Clinical data extraction

Three pathology providers (Clinipath Pathology, Australian Clinical Laboratories and Western Diagnostic Pathology) extracted the most recent blood test result prior to the CCTA scan for each patient, and these databases were merged to form “Database 1” (linked by their Medicare ID). Sex, medical and cardiac history (smoking status, diabetes diagnosis and previous CVD diagnosis), current medications, and at least three systolic blood pressure readings (mmHg) taken during the CCTA visit were obtained from three patient questionnaire forms in PRC records (see Supplemental Fig. 1). This was transcribed into “Database 3” using a combination of manual imputation for the handwritten information, and NLP for the tick-box information (performed by Max Kelsen Pty Ltd., see Supplemental Fig. 2). These identifiable databases were merged on a patient-level (linked by a randomly generated study ID) into an SPSS datafile, and then all identifying information was destroyed after manual verification and data cleaning (merging duplicate cases into single patient rows). Only the Principal Investigator and honors student had access to the identifiable information (protected by a16-character alphanumeric password) and take responsibility for its integrity, and additional researchers only had access to the de-identified master database. See Supplemental Fig. 3.

### 2.4 Cardiovascular risk scores

Each medical record was examined for lipid-lowering medications, as use of these medications can cause underestimation of CVRS [14]. When present, LDL adjustment was undertaken using composite scores from population data on each dose and type of statin [15,16], and corrected according to The Dutch Lipid Clinic Network Score [17] (see Supplemental Fig. 4). For CVRS calculations, the measured total cholesterol was used in patients not taking lipid-lowering therapy, and adjusted LDL concentration was used to calculate the total cholesterol used for those taking lipid-lowering therapy.

A CVRS was calculated for each eligible individual using the Australian Absolute Cardiovascular Risk Calculator (online Framingham-based CVRS calculator modified for the Australian population, see Supplemental Fig. 5), using information on the individual's sex, age, systolic blood pressure, diabetes and smoking status, total cholesterol, and HDL cholesterol levels [12]. Risk scores were classified as high risk (>15 %), moderate risk (10–15 %), low risk (1–9 %) or “zero” risk (>1 %) of experiencing a cardiovascular event in the next five years, with a maximum of 35 % [12]. A binary categorical variable was created with a zero-low risk score (≤9 %) and moderate-high risk score (>9 %).

### 2.5 Risk factor count

The number of traditional risk factors for each individual was counted and categorized as low (0–2), moderate (3–4) or high (5–6). Risk factors included diabetes, diagnosed hypertension (≥140 mmHg systolic blood pressure), current smoking status or having ceased within the last 12 months, male sex, age 35 to 74 years old at the time of the CCTA scan, and hypercholesterolemia (total cholesterol level of ≥5.5 mmol/L) [18]. Due to the possibility of a white-coat effect influencing measured blood pressure readings [19], only diagnosed hypertension was used as a risk factor.

### 2.6 Statistical analysis

All analysis was performed with IBM SPSS, version 28.0.1.1, and statistical significance was set at *p* < 0.05. Normality was assessed using the Shapiro-Wilk statistic, and the distribution of CVRS was compared in individuals with and without atherosclerosis through the independent *t*-test or Mann-Whitney *U* test (95 % CI), as appropriate. Non-parametric continuous variables were reported as median and interquartile range (25th percentile, 75th percentile), and categorical variables were reported as frequencies and percentages.

The primary analysis of this study was to determine the predictive accuracy of risk scoring in detecting pre-clinical CAD. Degree of atherosclerosis was compared to CVRS using the Fisher-Freeman-Halton or Monte-Carlo tests (95 % CI) to determine the significance of the relationship, and a weighted Cohen's kappa analysis for the level of agreement. The same analysis was undertaken to compare degree of atherosclerosis and risk factor count, and to compare CVRS to plaque extent, stenosis severity and CACS.

The proportion of the cohort with a match, and mismatch, between their CAD and CCTA results was determined to calculate the percentage of overestimation and underestimation. A correct match was defined as any direct match between the categories of CVRS and degree of atherosclerosis (e.g., low plaque-burden and low risk) and a mismatch was any other combination (e.g., extensive plaque-burden and low risk). The relationship strength was visualized by relationship maps and statistically tested. Proportions of the cohort with or without atherosclerosis, and with a moderate-high (>9 %) or zero-low (≤9 %) CVRS, was determined to calculate the sensitivity and specificity of the calculator and presented in a scaled rectangular diagram (personal communication, Roger Marshall, PhD, custom srd software, 2023). With CCTA as the reference group, the potential to improve classification of patients at non-high-risk using CVRS was examined by calculating the proportion of individuals who would be reclassified as high risk.

The risk calculator accounted for the potential confounding variables of sex and age, and males and females were analyzed separately. For a sensitivity analysis, variables were compared between the groups included and excluded from analysis. An independent *t*-test or Mann-Whitney *U* test was done for age, and patient frequencies were compared for the categorical variables of gender, presence of atherosclerosis and degree of atherosclerosis.

## 3 Results

### 3.1 Study population

**Fig. 1:**
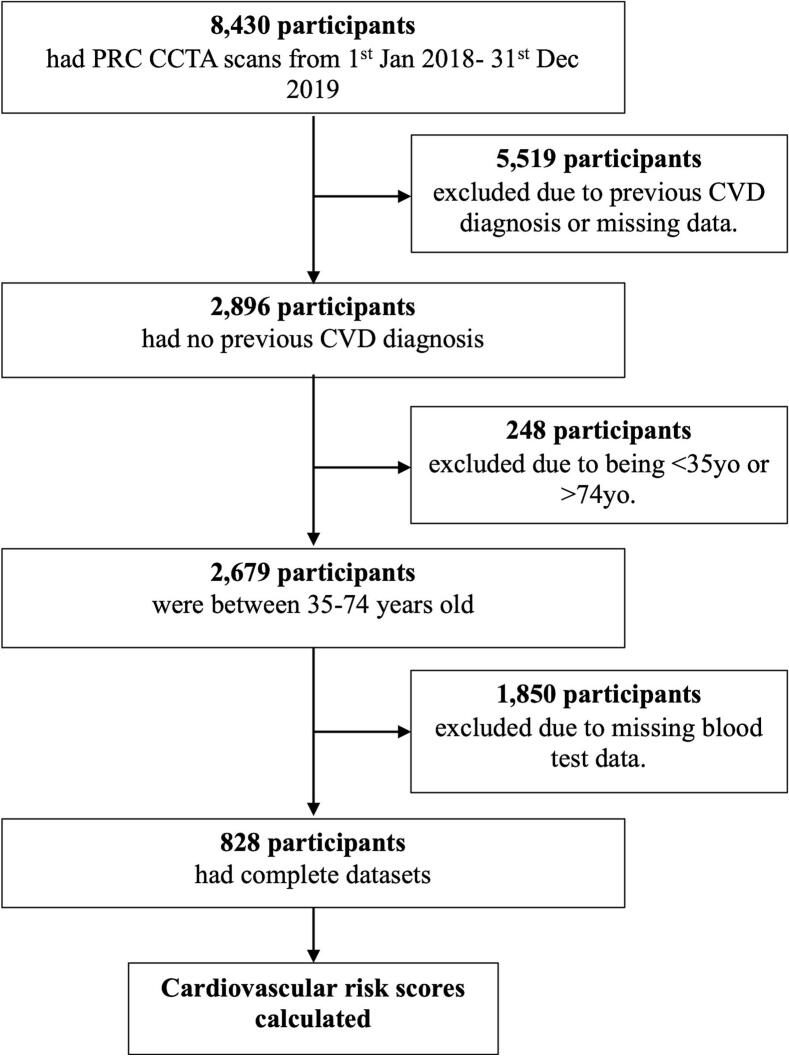
Final cohort extraction. PRC indicates Perth Radiological Clinic; CCTA indicates coronary computed tomography angiography; CVD indicates cardiovascular disease.

The study flow chart is summarized in Fig. 1. 828 patients met the inclusion criteria for this study. Of the total cohort of 8430 patients, 5519 had known CVD diagnosis or symptoms suggestive of CAD, were being evaluated for non-coronary cardiac surgery, or were missing at least one patient questionnaire form. A further 248 participants were outside of the required age range for CVRS calculation, and 1850 had at least one missing required blood measurement required. Another one patient was excluded because of the absence of a recorded CACS. Therefore, the analysis cohort consisted of 828 asymptomatic individuals with no prior history of CAD and sufficient data to calculate their CVRS.

### 3.2 Cohort characteristics

Cohort characteristics are summarized in Table 1. The median age was 58.6 years (IQR = 52.0, 65.3), with 358 (43.2 %) females and 470 (56.8 %) males. Low CVRS (1–9 %) was the most common subgroup of calculated 5-year risk (239 (50.9 %) males and 244 (68.2 %) females). Atherosclerosis to any extent was identified in 78.5 % (369) of males and 50.0 % (179) of females. A non-zero CACS was found in 386 (46.6 %) patients in the whole cohort (66.8 % male), with a median Agatston Unit of 2 (IQR = 0, 112) for males and 0 (IQR = 0, 15) for females. At the time of the CCTA, 144 (17.4 %) patients were prescribed lipid-lowering medication.

**Table 1:** Cohort clinical characteristics.

	Males (*n* = 470)	Females (*n* = 358)
Age, y, median (IQR)	57.35 (5, 18)	60.18 (3,11)
Hypertension, n (%)	183 (38.9 %)	140 (39.1 %)
Hypercholesterolemia, n (%)	212 (45.1 %)	120 (33.5 %)
Diabetes, n (%)	38 (8.1 %)	31 (8.7 %)
Smoking, n (%)	193 (41.1 %)	120 (33.2 %)
Blood pressure (mmHg), mean (SD)	131.84 (15.45)	130.64 (17.94)
Total cholesterol (mmol/L), mean (SD)	5.90 (1.64)	6.03 (1.53)
HDL cholesterol (mmol/L), mean (SD)	1.33 (0.32)	1.67 (0.46)
Risk factor count^a^, n (%)		
Low	82 (17.4 %)	216 (60.3 %)
Moderate	344 (73.2 %)	139 (38.8 %)
High	44 (9.4 %)	3 (0.8 %)
Cardiovascular risk score (%), median (IQR)	9 (5, 18)	5 (3, 11)
Cardiovascular risk score category^b^, n (%)		
Zero	1 (0.2 %)	12 (3.3 %)
Low	239 (51.0 %)	244 (68.0 %)
Moderate	84 (17.9 %)	29 (8.1 %)
High	145 (30.9 %)	74 (20.6 %)
Degree of atherosclerosis category, n (%)		
Low plaque-burden	147 (31.3 %)	72 (20.1 %)
Moderate plaque-burden	85 (18.1 %)	39 (10.9 %)
Extensive plaque-burden	137 (29.1 %)	68 (19.0 %)
No atherosclerosis	101 (21.5 %)	179 (50.0 %)

IQR indicates interquartile range, expressed as 25th percentile, 75th percentile; SD indicates standard deviation; HDL indicates high-density lipoprotein.

a Low (1–2 risk factors), moderate (3–4 risk factors), high (5–6 risk factors).

b “Zero” (<1 %), low (1–9 %), moderate (10–15 %) and high (>15 %) risk of experiencing a cardiovascular event in a 5-year period.

**Fig. 2:**
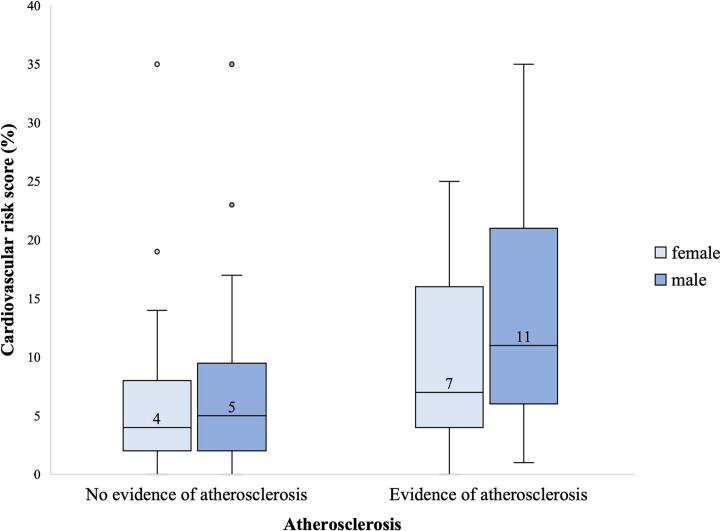
Distribution of cardiovascular risk score in individuals with and without atherosclerosis. Evidence of atherosclerosis indicates a detected abnormality in coronary computed tomography angiography and/or a non-zero coronary artery calcium score.

The continuous variable of CVRS was not normally distributed for males (W = 0.83, *p* < 0.001) or females (W = 0.69, p < 0.001), and homogeneity of variance was not assumed for males (F(1,468) = 8.69, *p* = 0.003) but was assumed for females (F(1,365) = 3.70, *p* = 0.055). The median risk score for males and females was, for the atherosclerosis group, 11 % and 7 %, and for the no atherosclerosis group, 5 % and 4 %, respectively. Significant differences in CVRS distribution were found between those with atherosclerosis and those without, in males (Mann-Whitney U 95 % CI: U = 26,533.00, n_1_ = 369, n_2_ = 101, *p* < 0.001) and females (U = 20,579.00, n_1_ = 179, n_2_ = 179, p < 0.001), shown in Fig. 2.

### 3.3 Mismatch analysis

**Fig. 3:**
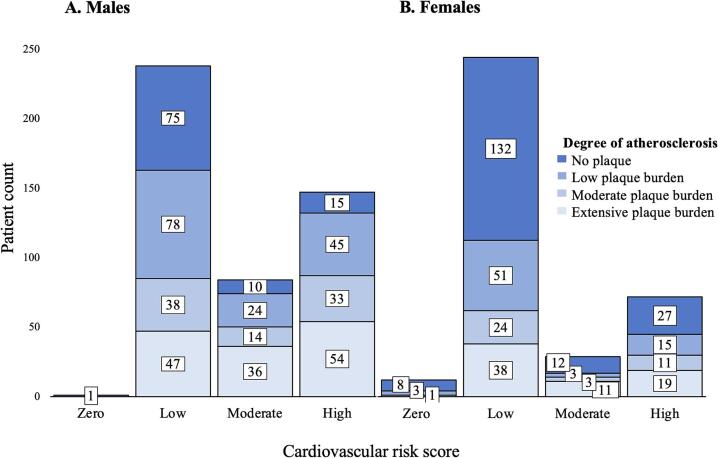
Proportion of individuals in each comparison group between cardiovascular risk score and degree of atherosclerosis categories. Category of risk score is “zero” (<1 %), low (1–9 %), moderate (10–15 %), high (>15 %) risk of experiencing a cardiovascular event in 5 years.

The mismatch between CVRS and degree of atherosclerosis is shown in Fig. 3. Of those with extensive atherosclerosis (137 males, 68 females), 47 (34.3 %) males and 38 (55.9 %) females had a low CVRS (1–9 %), and of those with no atherosclerosis (101 males and 179 females), 15 (14.9 %) and 27 (15.1 %) respectively were classified as high risk (>15 %). Additionally, of those with a low CVRS (238 males, 245 females), 47 (19.7 %) males and 38 (15.5 %) females had extensive atherosclerosis and of those with a high CVRS (147 males, 72 females), 15 (10.2 %) and 27 (37.5 %) respectively had no atherosclerosis. For males (*n* = 470), CVRS overestimation occurred in 43.0 % (202), underestimation in 25.7 % (121) and correct estimation of atherosclerosis risk in 31.3 % (147). In females (*n* = 358), overestimation occurred in 55.9 % (200), underestimation in 21.5 % (77) and correct estimation in 22.6 % (81).

A significant relationship (Monte-Carlo 95 % CI: male, *p* < 0.001; female, *p* = 0.024) but little to no agreement (Cohen's kappa: male, *κ* = 0.149; female, *κ* = 0.096) was found between degree of atherosclerosis and CVRS for both sexes, with low data accuracy (male, 2.2 %, *κ*^2^ = 0.022; female, 0.9 %, *κ*^2^ = 0.009). For CACS, a significant relationship (male, *p* < 0.001; female, *p* = 0.008) but little to no agreement (male, *κ* = 0.108; female, *κ* = 0.084) was found with CVRS for both sexes. Similarly for plaque extent, a significant relationship was found with CVRS for males (*p* < 0.001), but an insignificant relationship for females (*p* = 0.121), with little to no agreement for males (*κ* = 0.110) and females (*κ* = 0.080). Finally for stenosis severity, a significant relationship (male, p < 0.001; female, *p* = 0.012) but little to no agreement (male, *κ* = 0.035; female, *κ* = 0.030) was found with CVRS for both sexes.

**Fig. 4:**
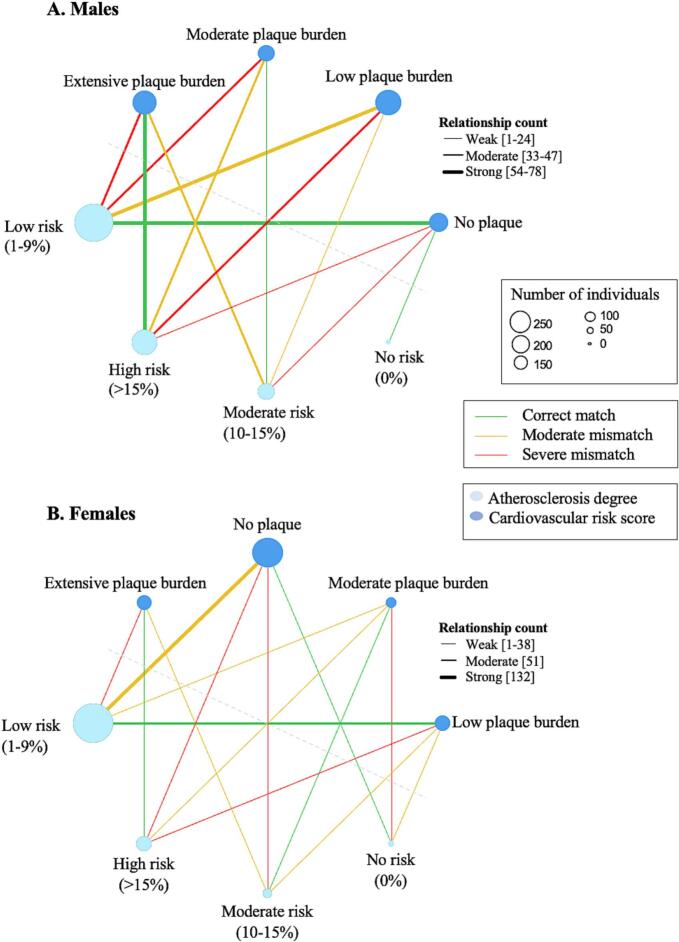
Relationship maps comparing degree of atherosclerosis categories and cardiovascular risk score. The red and orange lines represent a mismatch (red = severe, orange = not severe), and the green lines represent a match, between the categories. The line thickness represents the relationship strength (relationship count, the number of patients in that comparison group) and the circles represent categories of each variable, with the size correlating to patient count. (For interpretation of the references to color in this figure legend, the reader is referred to the web version of this article.)

**Fig. 5:**
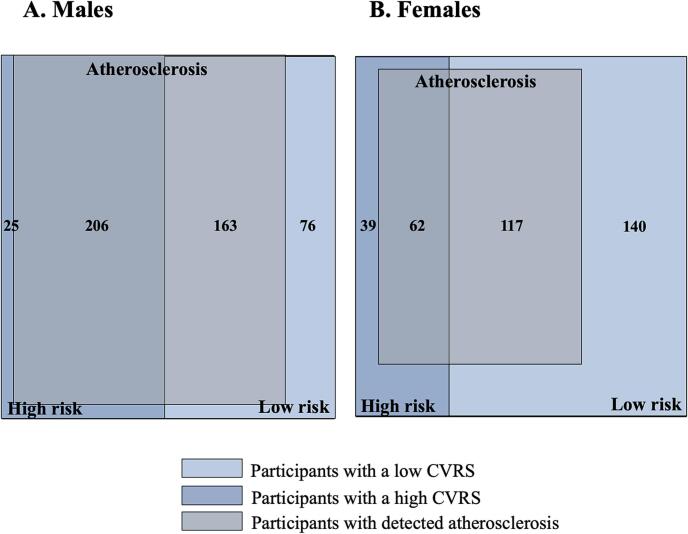
Proportions of individuals classified as high risk and low risk, and who had detected atherosclerosis. CVRS indicates cardiovascular risk score, and high-risk is moderate-high (>9 %) and low-risk is zero-low (≤9 %). Created using custom srd software (Roger Marshall, PhD).

The relationship between the degree of atherosclerosis and CVRS is shown in Fig. 4, with line thickness indicating relationship strength and color representing a match or mismatch. In males, a strong relationship was observed between low risk and no atherosclerosis as well as low burden atherosclerosis (count = 75, 78), and between extensive atherosclerosis and high risk (count = 54). However, a moderate relationship was observed between extensive atherosclerosis and low risk (count = 47) and between low burden atherosclerosis and high risk (count = 45). For females, the only strong relationship was between no atherosclerosis and low risk (count = 132). The relationship between low risk and extensive atherosclerosis (count = 38) and high risk and no atherosclerosis (count = 27) was stronger than between high risk and extensive atherosclerosis (count = 19). As shown in Fig. 5, for both sexes, there is a substantial proportion of low-risk individuals with demonstratable atherosclerosis. Of the 369 males and 179 females with atherosclerosis, 44.2 % (163) and 65.4 % (117) were classified as zero or low risk, respectively. The sensitivity of the risk calculator for males and females was determined to be 89.2 % and 61.4 %, and the specificity 31.8 % and 54.5 %, respectively.

Assuming the 147 males and 72 females with a high CVRS remain in this category even if CCTA did not demonstrate atherosclerosis, we calculated the potential for CCTA to reclassify the 239 males and 257 females with a CVRS of ≤9 % according to their actual observed plaque-burden. Identifying a moderate to high plaque-burden would reclassify 84 males (35.1 %) and 63 females (24.5 %) to a CVRS of >9 %. Identifying any atherosclerosis would reclassify 163 men (68.2 %) and 117 women (45.5 %) to a CVRS of >9 %.

### 3.4 Risk factor counting

**Fig. 6:**
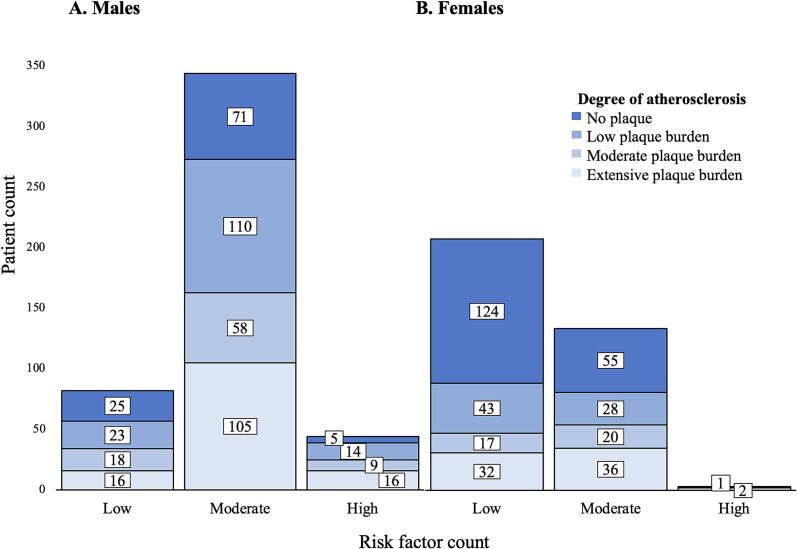
Comparison of risk factor count and degree of atherosclerosis categories. Risk factor count is categorized as low (1–2 risk factors), moderate (3–4 risk factors) and high (5–6 risk factors).

The risk factor count and degree of atherosclerosis mismatch is observed in Fig. 6. Of the 137 males and 68 females with extensive atherosclerosis, 16 (11.7 %) and 32 (47.1 %) respectively had only one or two risk factors. Of the 101 males and 179 females without atherosclerosis, 76 (75.2 %) and 55 (30.7 %) respectively had more than three risk factors. A non-significant relationship between risk factors and atherosclerosis was found for males (Monte-Carlo 95 % CI: *p* = 0.101) and a significant relationship for females (*p* < 0.001), but little to no agreement in both sexes (Cohen's kappa: male, *κ* = 0.027; female, *κ* = 0.044), indicating low data accuracy (male, 0.1 %, *κ*^2^ = 0.001; female, 0.2 %, *κ*^2^ = 0.002).

### 3.5 Sensitivity analysis

Of the total cohort with missing data (*n* = 5463, although all had CCTA and CACS data available), 2447 (44.8 %) were female and 3016 (55.2 %) were male, with a median age of 59.7 years (IQR = 52.4, 66.3). The age distribution was similar to the analysis cohort for both sexes (males, U = 748,426.00, *p* = 0.065; females, U = 466,714.50, *p* = 0.081). In this cohort, no atherosclerosis was reported in 1051 (43.0 %) females and 739 males (24.5 %).

## 4 Discussion

To our knowledge, this is the first study to demonstrate a substantial mismatch between predicted CVRS and the extent of CAD identified using CCTA in asymptomatic individuals with no prior CAD history. Although subclinical CAD was detected more frequently in those with higher risk scores, agreement between degree of atherosclerosis and CVRS was poor. Importantly, a low CVRS was calculated in 34 % of males and 56 % of females found to have extensive CAD. At least some mismatch was identified in 69 % of males and 77 % of females, highlighting that CVRS is not reliable in predicting CAD. This has important implications in clinical practice, particularly in apparently low risk individuals who may be falsely reassured against the presence of CAD.

Pen et al. [20] used a similar methodology to investigate the discordance between Framingham risk score (FRS) and CCTA-defined atherosclerosis burden, however most patients in their US-based cohort had chest pain or other symptoms suggestive of CAD. Consistent with our findings, they found a weak association between plaque-burden and FRS, with 47.6 % of low FRS-risk patients having detected atherosclerosis. Their analysis compared FRS to total plaque score (TPS) obtained by CCTA, and did not consider plaque location, stenosis severity or the number of affected arteries, as addressed in the present study. Most prior studies assessing CVRS reliability have investigated subsequent cardiovascular event rates [6,21] instead of the presence and burden of atherosclerosis in asymptomatic individuals. It is consistently demonstrated that untreated high-risk individuals have high event rates [22], highlighting the importance of aggressive risk factor reduction. Conversely, relatively few CV events occur in low-risk individuals, however due to the high proportion of apparently low-risk individuals in the community, the largest total number of events occur in this group [22]. This highlights a significant unmet need to identify those individuals not traditionally considered high-risk, but who will subsequently have a cardiovascular event [6,21]. Vernon et al. [23] reported that, of those hospitalized with an MI, 25 % did not have any traditional risk factors and had a higher mortality risk than those with at least one risk factor [5]. Similarly, we demonstrated that 12 % of males and 47 % of females with extensive atherosclerosis had only one or two traditional risk factors, with poor agreement between CVRS and degree of atherosclerosis for both sexes. Although better than chance alone, CVRS used in isolation would leave many non-high-risk patients with undetected CAD, including apparently low-risk individuals with extensive disease, potentially leading to a fatal coronary event. Additionally, we observed CVRS overestimation in approximately half of the cohort, leading to potential over-treatment in the absence of disease. This overestimation tendency has been reported by others [6,21,24], however we found a larger proportion of overestimated patients, suggesting that CVRS may have a more important role in prediction of a future cardiovascular event risk, than that of subclinical CAD. Therefore, mechanisms other than atherosclerosis may contribute to cardiovascular events, although identifying asymptomatic CAD may be seen as an opportunity to detect-and-treat prior a fatal event. Recent findings by Medina et al. [25] demonstrated reasonable performance of CVRS in predicting future cardiovascular events, with only 2.3 % of non-high-risk individuals experiencing an event, although CCTA or CACS were not examined in this study.

Outside of the potential benefits of CCTA, CACS has emerged as the single best predictor of subsequent coronary events in asymptomatic individuals [26,27]. Hoffman et al. [28] demonstrated that in those with a low CVRS (<6 %), 32 % of men and 23 % of women had a non-zero CACS. Further, Venkataraman et al. [7] identified 77 % of patients with CACS>100 that were classified as low-risk. Hence, CACS has been recommended to form part of standard cardiovascular risk assessments in middle aged and older non-high-risk individuals [29]. Beyond this, Hadamitzky et al. [30] reported that CCTA provides better prediction of future cardiovascular events than CVRS and reinforces that identification of subclinical atherosclerosis may have important prognostic implications in otherwise low-risk patients.

The mismatch between CVRS and presence and extent of CAD is likely explained by the combination of environmental influences [22] and non-traditional risk factors that can influence atherosclerosis development, such as additional lipoproteins not considered in CVRS [such as Lp(a)], genetic loci [31], psychosocial factors [32], and exercise and diet [29]. However, it is not currently viable to assess all potential non-traditional risk factors across a population. Similarly however, although CCTA may reveal the final atherosclerotic result of exposure to risk factors, it is also not feasible to deploy such imaging across a population of apparently low-risk individuals. Additional research is needed to identify which apparently low-risk individuals would benefit from a more intensive search for atherosclerosis including CCTA.

### 4.1 Limitations

It is important to note that we excluded patients with known CVD and symptoms suggestive of CAD. In addition, this study has limitations to its applicability to a broader population. A selection bias is likely since patients were referred for CCTA presumably because of clinical uncertainty by the referring doctor. Further, since CCTA for a cardiovascular risk assessment in asymptomatic individuals is not Medicare-funded in Australia [2], the scan cost could be a potential source of bias. While the proportion of individuals with atherosclerosis in this cohort is likely larger than that of the general population, the study still illustrates the limitations of current CVRS techniques compared with the gold standard of CCTA for atherosclerosis assessment. Finally, we cannot exclude the possibility that the missing data, due to the retrospective nature of this study, may have influenced the measured outcomes. However, a sensitivity analysis confirmed that the excluded sample had similar characteristics to the final analysis cohort.

## 5 Conclusions

In asymptomatic individuals with no CVD history undergoing CCTA, CVRS does not reliably predict the presence or extent of CAD. A mismatch between CVRS and the degree of CAD (identified by CCTA and CACS) was found in majority of the cohort, and extensive CAD was observed in a significant proportion of individuals who otherwise appeared to be at low-risk. This highlights a potential role for CCTA in non-high-risk individuals with clinical uncertainty, where reclassification of risk based on cardiovascular imaging may influence treatment decisions (i.e., initiation of lipid-lowering therapies). Randomized prospective studies are required to address whether such an approach will improve outcomes in Australia.

## CRediT authorship contribution statement

**Emma Playford:** Data curation, Formal analysis, Investigation, Methodology, Software, Visualization, Writing – original draft, Writing – review & editing. **Simon Stewart:** Conceptualization, Methodology, Supervision, Writing – review & editing. **Gerard Hoyne:** Methodology, Supervision, Writing – review & editing. **Geoff Strange:** Writing – review & editing. **Girish Dwivedi:** Conceptualization, Writing – review & editing. **Christian Hamilton-Craig:** Writing – review & editing. **Gemma Figtree:** Writing – review & editing. **David Playford:** Conceptualization, Data curation, Funding acquisition, Methodology, Project administration, Resources, Writing – review & editing.

## Funding

This study was funded by an Investigator-Initiated Grant from the Perth Radiological Clinic Foundation.

## Declaration of competing interest

None.

## Appendix A Supplementary data

Supplementary material Image 1
