# Supplementary material for: Comparing predictive risk to actual presence of coronary atherosclerosis on coronary computed tomography angiography
Source: Am Heart J Plus. 2024 Dec 6;49:100493. doi: 10.1016/j.ahjo.2024.100493 (PMC11698937; doi:10.1016/j.ahjo.2024.100493)

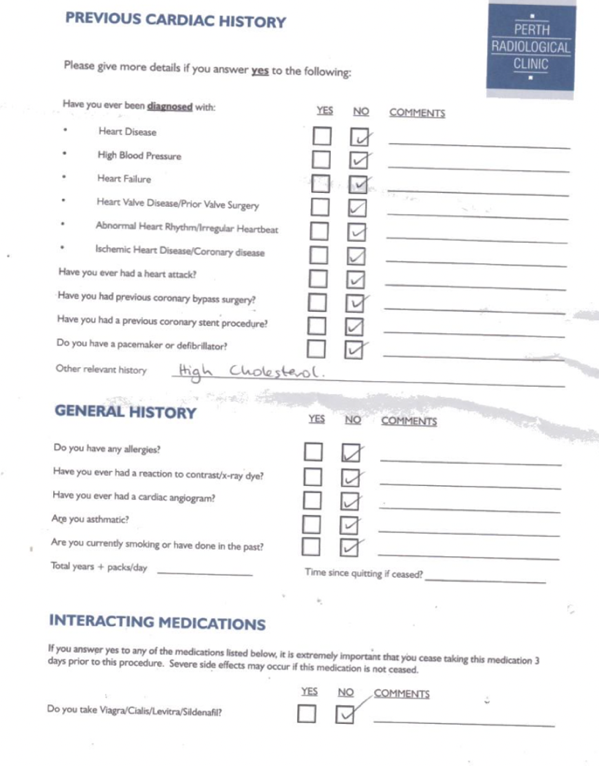

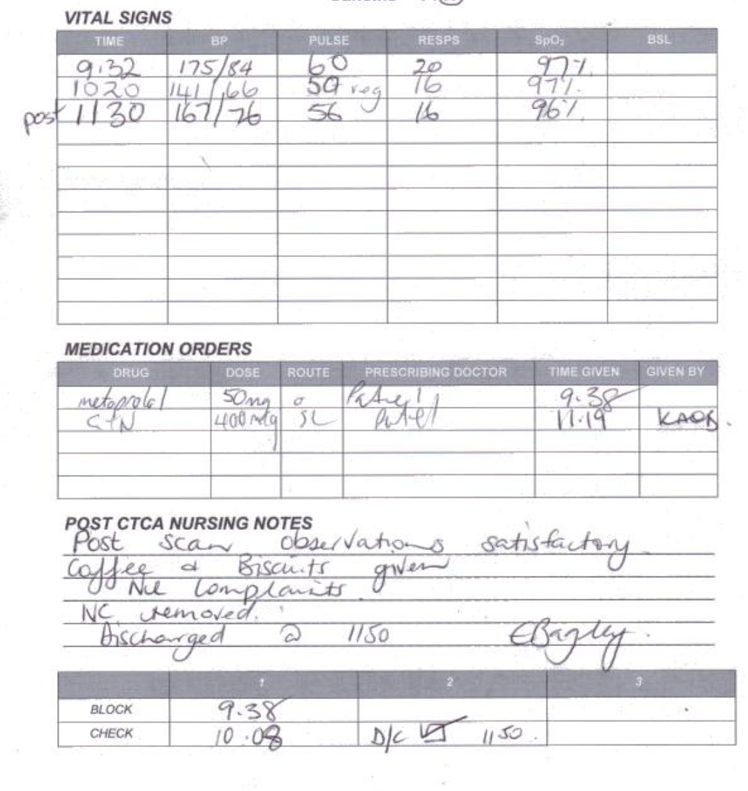
**Supplementary Material**


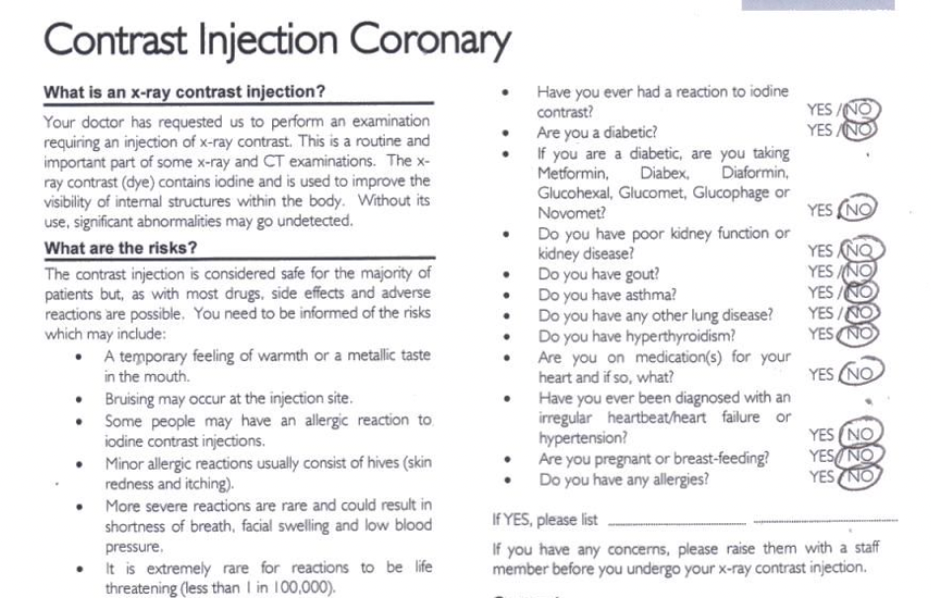


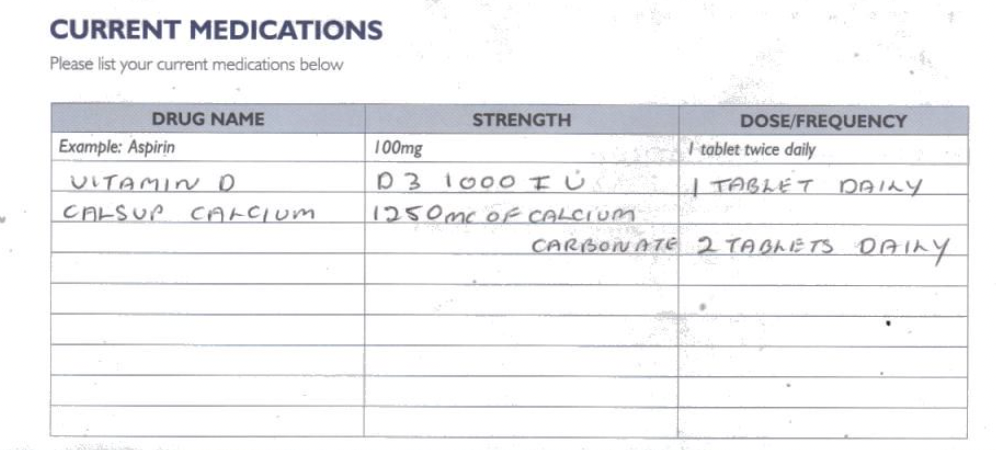


**Supplemental Figure 1.** Examples of PRC Patient Questionnaire Forms.


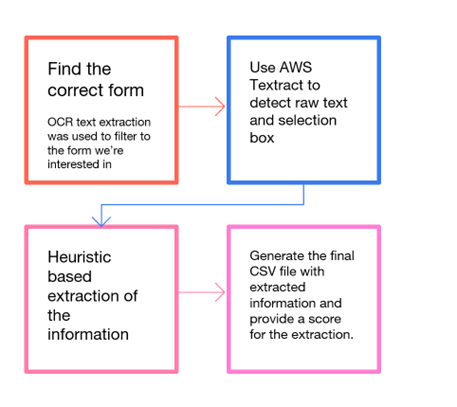


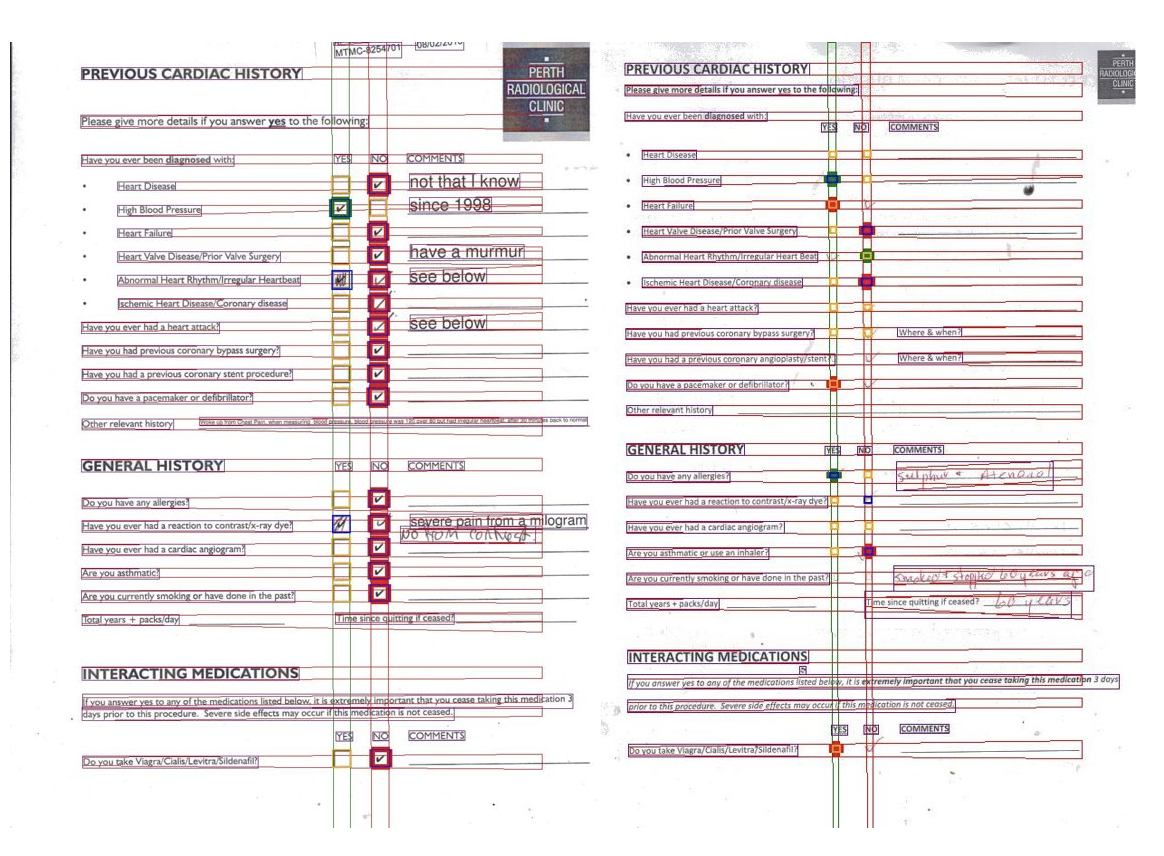


**Supplemental Figure 2.** Max Kelsen Pty Ltd Methodology for Extraction of Tick-Box Information through AI Natural Language Processing. OCR indicates Optical Character Recognition; AWS indicates Amazon Web Services; CSV indicates Comma Separated Values.

**Australian Clinical Laboratory, Clinipath pathology and Western Diagnostics pathology**

Add lipid results into database

All other datapoints removed except Study ID and lipid results

**Database 3**

PRC Patient questionnaire data

Password protected and upload to **One Drive** by PRC

(Only shared to the principal investigator)

Patient information in databases:

**Database 2a:**

DOB, CCTA scan date, Medicare ID number, COMRAD visit number

**Database 2b:**

COMRAD visit number and CCTA results

**Database 3:**

Sex, medical and cardiac history, medications, blood pressure and COMRAD visit number

New **Study ID** created with random number generator and COMRAD visit number removed

**PRC Database 2b**

CCTA and CACS report data from PRC

**PRC Database 2a**

Identifiable patient data

Password protected and uploaded to **One Drive** by pathology provider

Merge the 3 pathology databases

**Database 1**

Pathology results

Re-insert COMRAD visit number

**Database 1**

Pathology data, Study ID, COMRAD visit number

Merge database 1 and 2b

**Master database**

Merge database 3 and merged 1 and 2b

Remove and destroy all identifiable data (COMRAD visit number, Medicare ID number, DOB)

Secure storage and documentation of use

**Supplemental Figure 3.** Flowchart of the data extraction process. PRC indicates Perth Radiological Clinic; CCTA indicates coronary computed tomography angiography; COMRAD is the radiology information solutions provider used by PRC.


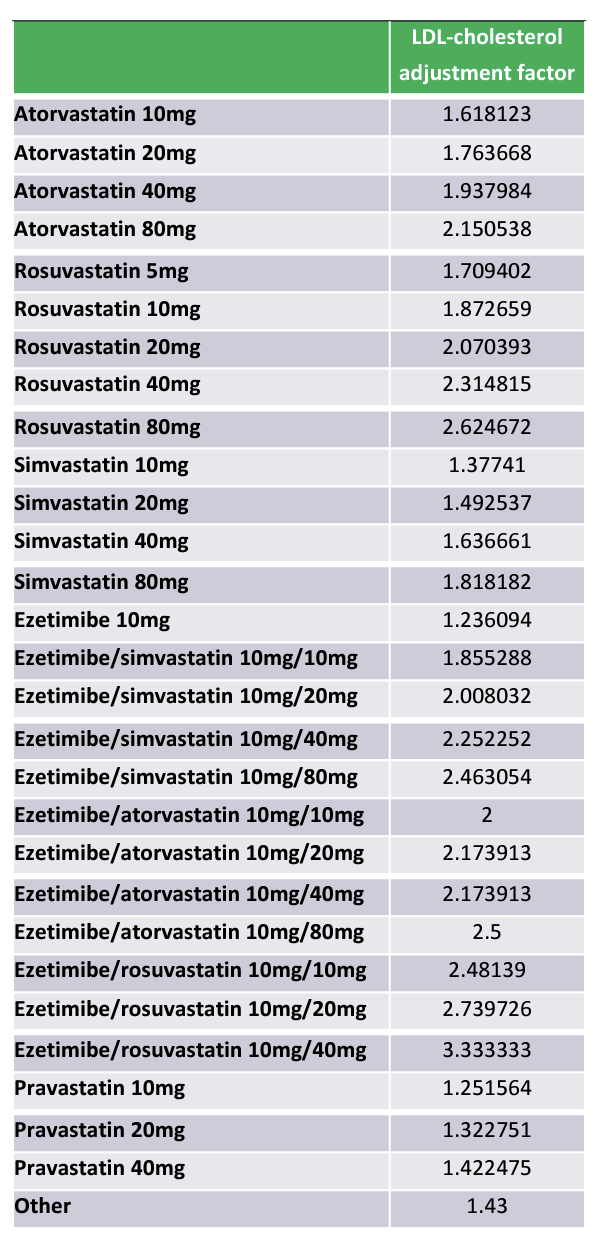
Dutch Lipid Clinic Network Score manual calculation^17^:

Proportion of population^15,16^

- Atorvastatin 44.6% (20mg)
- Rosuvastatin 35.4% (10mg)
- Simvastatin 16.7% (40mg)
- Pravastatin 3.3% (40mg)
- Ezetimibe (10mg) + Atorvastatin (20mg) 3%
- Ezetimibe (10mg) + Rosuvastatin (10mg) 7.6%
- Ezetimibe (10mg) + Simvastatin (40mg) 89.4%

**Composite scores=**

**Proportion of population on drug x Adjustment factor**

- Statin

Atorvastatin 20mg =1.763

Rosuvastatin 10mg =1.87

Simvastatin 40mg =1.637

Pravastatin 40mg =1.422

0.446x1.763 + 0.354x1.87 + 0.167x1.637 + 0.033x1.422

= **1.769**

- Ezetimibe (10mg) + Statin combination

+ Atorvastatin 20mg =2.17

+ Rosuvastatin 10mg =2.48

+ Simvastatin 40mg =2.25 
0.894x2.25 + 0.076x2.48 + 0.03x2.17

= **2.26**

**Supplemental Figure 4.** Calculations for LDL Cholesterol Levels Adjusted for Lipid-Lowering Medication.

**
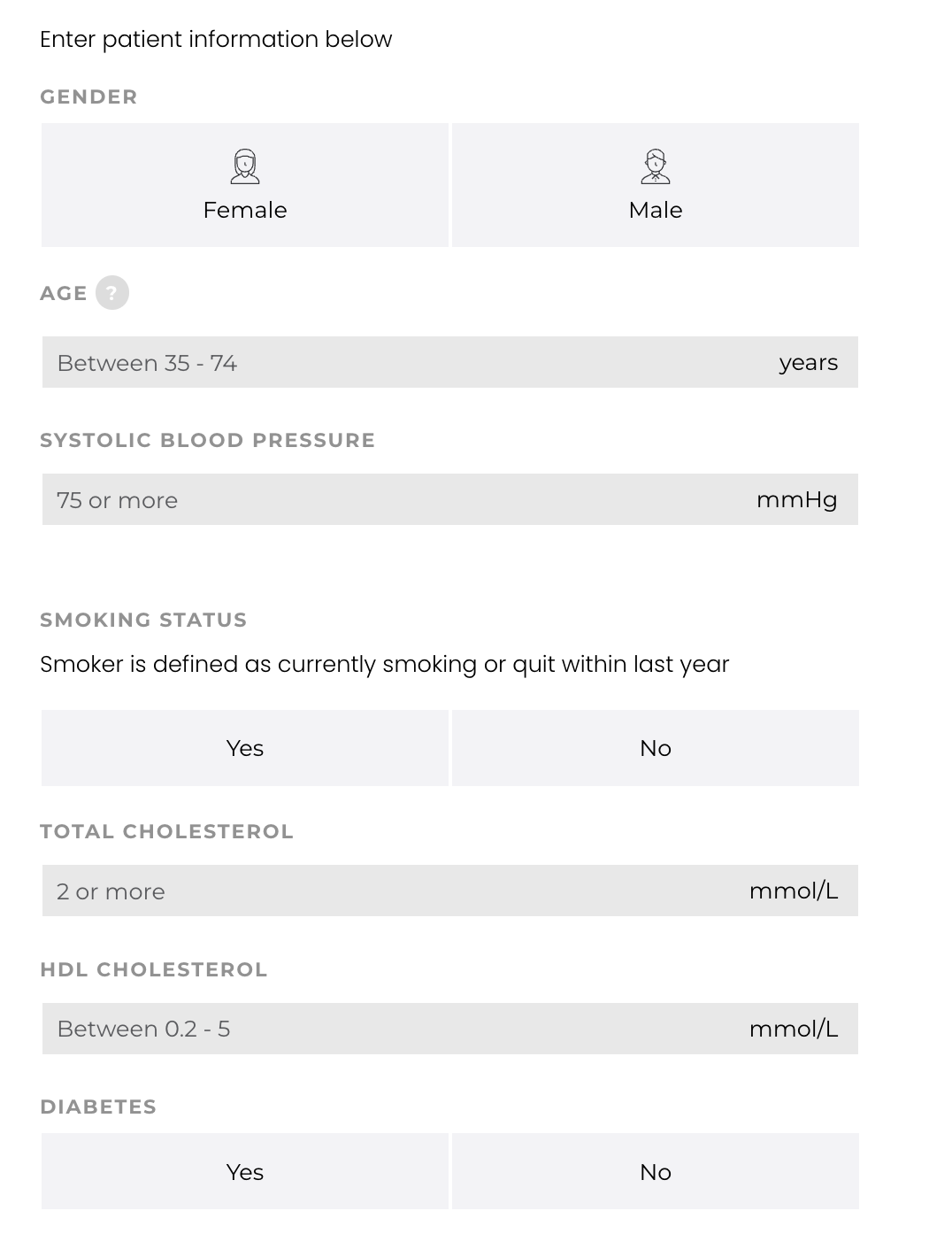
**

**
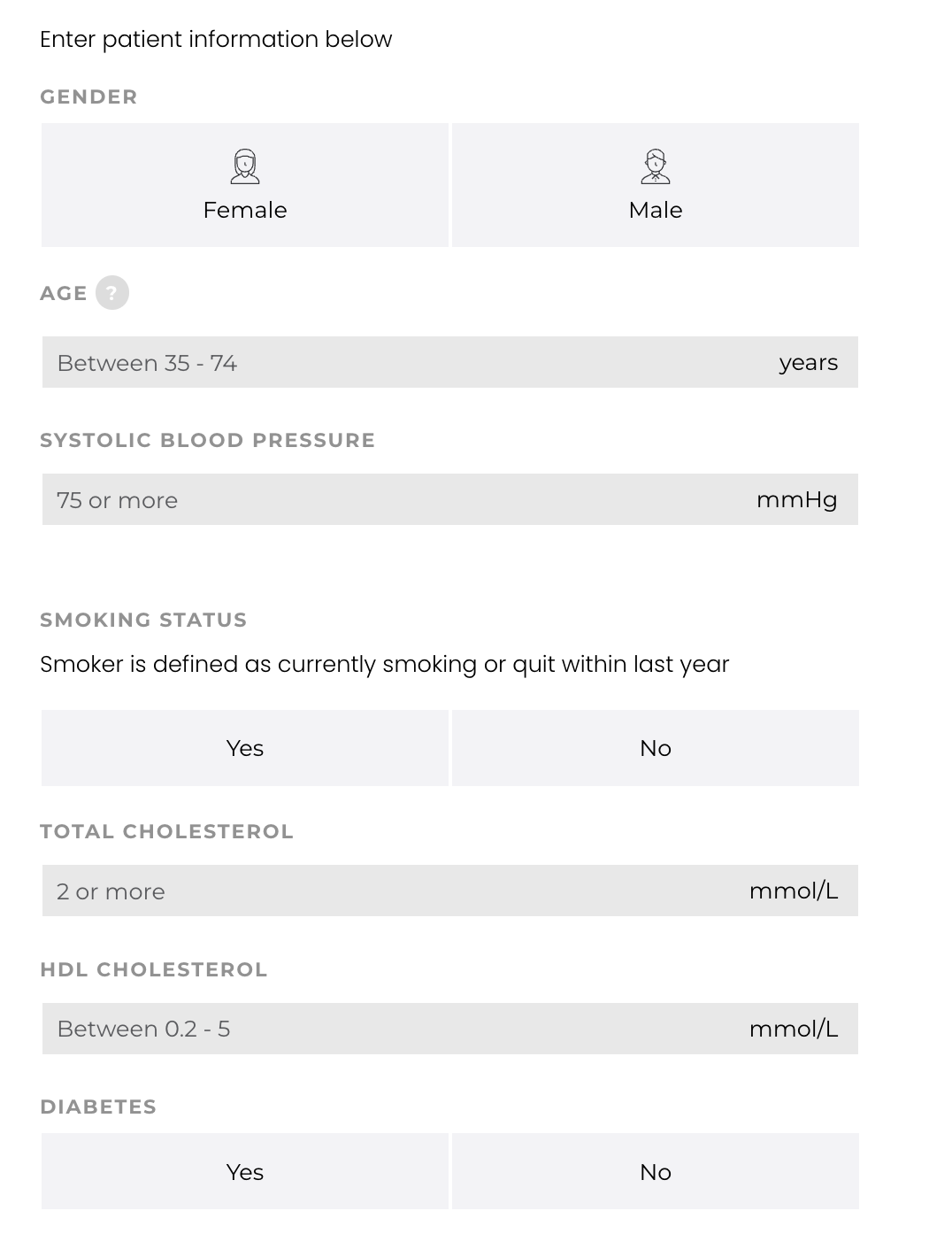
**

**Supplemental Figure 5.** Australian Absolute Cardiovascular Risk Calculator.^12^

**Supplemental Table 1.** Raw Crosstabulation Table Comparing Cardiovascular Risk Score Category to Degree of Atherosclerosis for Males and Females.

|  | **Degree of Atherosclerosis (plaque-burden)** | | | | | | | | | |
| --- | --- | --- | --- | --- | --- | --- | --- | --- | --- | --- |
|  | None | | Low | | Moderate | | Extensive | | **Total** | |
|  | Males | Females | Males | Females | Males | Females | Males | Females | Males | Females |
| No risk (0%) | 1 | 8 | 0 | 3 | 0 | 1 | 0 | 0 | 1 | 12 |
| Low risk (1-9%) | 75 | 132 | 78 | 51 | 38 | 24 | 47 | 38 | 238 | 245 |
| Moderate risk (10-15%) | 10 | 12 | 24 | 3 | 14 | 3 | 36 | 11 | 84 | 29 |
| High risk (>15%) | 15 | 27 | 45 | 15 | 33 | 11 | 54 | 19 | 147 | 72 |
| **Total** | 101 | 179 | 147 | 72 | 85 | 39 | 137 | 68 | 470 | 358 |

Key:


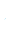
Overestimation



Underestimation



Correctly estimated



**Supplemental Table 2.** Raw Crosstabulation Table Comparing Evidence of Atherosclerosis to a Low or Moderate-High Risk Score for Males and Females.

|  | **Evidence of atherosclerosis** | | | | | |
| --- | --- | --- | --- | --- | --- | --- |
|  | None | | Present | | **Total** | |
|  | Males | Females | Males | Females | Males | Females |
| Low risk (≤9%) | 76 | 140 | 163 | 117 | 239 | 257 |
| Moderate- High risk (>9%) | 25 | 39 | 206 | 62 | 231 | 101 |
| **Total** | 101 | 179 | 369 | 179 | 470 | 358 |

 Key:


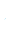
True negative



False negative



True positive



False positive



**Supplemental Table 3.** Raw Crosstabulation Table Comparing Risk Factor Count Category to Degree of Atherosclerosis for Males and Females.

|  | **Degree of Atherosclerosis (plaque-burden)** | | | | | | | | | |
| --- | --- | --- | --- | --- | --- | --- | --- | --- | --- | --- |
|  | None | | Low | | Moderate | | Extensive | | **Total** | |
|  | Males | Females | Males | Females | Males | Females | Males | Females | Males | Females |
| Low (1-2) | 25 | 124 | 23 | 43 | 18 | 17 | 16 | 32 | 82 | 216 |
| Moderate (3-4) | 71 | 55 | 110 | 28 | 58 | 20 | 105 | 36 | 344 | 139 |
| High (5-6) | 5 | 0 | 14 | 1 | 9 | 2 | 16 | 0 | 44 | 3 |
| **Total** | 101 | 179 | 147 | 72 | 85 | 39 | 137 | 68 | 470 | 358 |

Key:


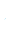
Overestimation



Underestimation



Correctly estimated


Supplement: Supplementary file 1 — Supplementary material [file mmc1.docx]
